# Why High‐Quality Guidelines Matter in Gastroenterology—And How UEG Is Leading the Way

**DOI:** 10.1002/ueg2.70079

**Published:** 2025-07-17

**Authors:** Brigida Barberio, Jordi Serra, Iago Rodríguez‐Lago

**Affiliations:** ^1^ Department of Surgery Oncology and Gastroenterology (DISCOG) Gastroenterology Unit University of Padova‐Azienda Ospedaliera di Padova Padova Italy; ^2^ Digestive System Research Unit Vall d’Hebron University Hospital Barcelona Spain; ^3^ Centro de Investigación Biomédica en Red de Enfermedades Hepáticas y Digestivas (Ciberehd) Barcelona Spain; ^4^ Gastroenterology Department Hospital Universitario de Galdakao Biobizkaia Health Research Institute Galdakao Spain

In an increasingly complex and fast‐evolving medical landscape, clinical guidelines have become essential tools for promoting effective, safe, and equitable care [1]. This is especially true in gastroenterology. From inflammatory bowel disease to gastrointestinal oncology, from gut‐brain disorders to the rise of artificial intelligence in endoscopy, change is rapid—and guidelines offer direction. High‐quality clinical guidelines provide a reliable reference built on the best available evidence and expert judgment [2]. They create a shared framework within which individualized decisions can be tailored to each patient’s needs. In doing so, they reduce heterogeneity, promote safety, and directly contribute to better outcomes and patient satisfaction [1].

For patients, guidelines help clarify complex treatment landscapes. They offer accessible, evidence‐informed summaries of risks and benefits, empowering individuals to make informed, shared decisions. This ensures that care is both effective and aligned with personal values [1].

For clinicians, guidelines are a critical resource. They distill scientific evidence into practical recommendations, helping avoid ineffective or harmful interventions and enhancing efficiency. They also foster a common language across countries and institutions, supporting harmonization of care. Moreover, they serve as valuable educational tools for trainees and early‐career professionals [3].

To be truly impactful, guidelines must be developed through a rigorous, validated methodology. They should incorporate all available evidence and provide actionable recommendations that are feasible across different settings and patient populations. Recognizing this, United European Gastroenterology (UEG) has taken a central role. In 2019, UEG established the Quality of Care task force—now a permanent committee—to improve healthcare standards across Europe. Among its main objectives is the promotion of trustworthy, evidence‐based guidelines.

UEG has introduced the Activity Grant programme to support guideline development and has defined strict methodological and structural standards. All endorsed guidelines must comply with AGREE II, as this guideline appraisal tool provides a framework of six domains that assesses the methodological quality and transparency of clinical practice guidelines [4, 5]. Regarding the professionals involved, each proposal must include a certified methodologist, who ensures methodological rigor and transparency—from question formulation through evidence appraisal to the drafting of recommendations. Also, the GRADE approach is mandatory, and the lead of the project must complete UEG’s dedicated training course. Concerning the approach to the content and structure of the guideline, clinical questions should be structured using the PICO format (Population, Intervention, Comparator, Outcome), which promotes clarity, focus, and reproducibility in evidence synthesis [6].

Inclusiveness, feasibility, and clinical relevance are also essential. UEG encourages gender and regional diversity within working groups, as well as involvement of junior professionals. Patient representatives and primary care physicians are also valuable contributors, helping to ensure guidelines are accessible and grounded in real‐world practice.

Once developed, the whole UEG structure plays a crucial role in dissemination so this ensures that the work can reach the whole Gastroenterology community. Guidelines are shared through UEG Journal and digital platforms, with continued efforts to support their uptake through funding and training. Over time, this has help UEG Journal to become a hub for high‐impact clinical guidelines, advancing digestive health across Europe and beyond.

Looking ahead, guidelines should also evolve to meet the needs of a rapidly changing world. “Living guidelines”, updated continuously as new evidence emerges, are already being piloted in fast‐moving areas such as artificial intelligence in gastroenterology [7]. Visual summaries, concise formats, and mobile tools are enhancing usability in clinical settings [8]. Cross‐society collaboration is also growing, fostering shared standards and reducing fragmentation, also leading to recommendations that are closer to our clinical practice and useful for different professionals involved in patient care. Transparency, accessibility, and patient involvement will be key pillars for the future [9].

In conclusion, clinical guidelines are vital tools at the intersection of science, practice, and patient‐centered care. UEG's commitment to supporting high‐quality guideline development is not only a service to clinicians, but a testament to its mission of improving digestive health across Europe. Through its grants, training, and publication platforms, UEG is driving progress in gastroenterology—one high‐quality guideline at a time.

## Conflicts of Interest

The authors declare no conflicts of interest.

## Data Availability

Data sharing not applicable to this article as no datasets were generated or analyzed during the current study.
